# BioJava-ModFinder: identification of protein modifications in 3D structures from the Protein Data Bank

**DOI:** 10.1093/bioinformatics/btx101

**Published:** 2017-02-17

**Authors:** Jianjiong Gao, Andreas Prlić, Chunxiao Bi, Wolfgang F Bluhm, Dimitris Dimitropoulos, Dong Xu, Philip E Bourne, Peter W Rose

**Affiliations:** 1 Department of Computer Science and C.S. Bond Life Sciences Center, University of Missouri, Columbia, MO, USA; 2 RCSB Protein Data Bank, San Diego Supercomputer Center, University of California San Diego, La Jolla, CA, USA; 3 Skaggs School of Pharmacy and Pharmaceutical Sciences, University of California San Diego, La Jolla, CA, USA

## Abstract

**Summary:**

We developed a new software tool, BioJava-ModFinder, for identifying protein modifications observed in 3D structures archived in the Protein Data Bank (PDB). Information on more than 400 types of protein modifications were collected and curated from annotations in PDB, RESID, and PSI-MOD. We divided these modifications into three categories: modified residues, attachment modifications, and cross-links. We have developed a systematic method to identify these modifications in 3D protein structures. We have integrated this package with the RCSB PDB web application and added protein modification annotations to the sequence diagram and structure display. By scanning all 3D structures in the PDB using BioJava-ModFinder, we identified more than 30 000 structures with protein modifications, which can be searched, browsed, and visualized on the RCSB PDB website.

**Availability and Implementation:**

BioJava-ModFinder is available as open source (LGPL license) at (https://github.com/biojava/biojava/tree/master/biojava-modfinder). The RCSB PDB can be accessed at http://www.rcsb.org.

## 1 Introduction

Chemical modifications of proteins, such as glycosylation, phosphorylation, sulfation, and acetylation, are ubiquitous and dynamic processes in living cells, modulating diverse protein functions. Although commonly referred to as post-translational modifications (PTM), protein modifications may occur before, during, or after protein synthesis (Farriol-Mathis *et al.*, 2004). Analysis of these modifications is particularly important for understanding protein functions in health and disease—the initiation and progression of many human conditions such as cancer, diabetes, and neurodegenerative diseases are related to specific patterns of protein modifications.

Protein modifications are annotated in the UniProtKB sequence database based on a variety of data including descriptions in the literature, annotations in specific databases, observations in 3D structures, inferred from related proteins, or predicted using rule-based systems (Farriol-Mathis *et al.*, 2004). Here we are interested in comprehensively annotating protein modifications that are present in the 3D structures archived in the Protein Data Bank (PDB) (Berman *et al.*, 2000).

We developed a new BioJava (Prlić *et al.*, 2012) package, ModFinder, for identification of protein modifications in 3D structures. Information about various modifications was collected from annotations in the PDB, the RESID database of protein modifications (Garavelli, 2004), and the PSI-MOD ontology (Montecchi-Palazzi *et al.*, 2008). These modifications were classified into three categories: modified residues, attachment modifications, and cross-links. Different strategies were developed to identify these modifications in the PDB. We identified more than 30 000 structures with one or more protein modifications and have annotated these on the sequence diagrams displayed on the RCSB PDB website.

## 2 Implementation

### 2.1 Classification of protein modifications

Protein modifications can be classified in various ways. We classified protein modifications in the PDB into three categories: (i) modified residues: which include modification of amino acid residues with small chemical groups and are treated as single residues in the PDB (e.g. hydroxyproline), (ii) attachment modifications: where amino acid residue(s) in the protein attach to larger chemical groups, ions or ligands and are presented in the PDB as a separate chemical component linked to a standard amino acid (e.g. glycan attached to an asparagine), and (iii) cross-links: where two or more amino acid residues are linked through non-peptide bonding interactions (e.g. disulfide bond).

### 2.2 Data collection

We collected data for more than 400 protein modifications from sources including PDB, RESID, and PSI-MOD, and stored them in a customized XML file, including the following information: modification identifiers and names in RESID and PSI-MOD, brief description from PSI-MOD, systematic name from RESID, category, keywords, PDB names of the amino acids and/or ions, ligands and other chemical components involved, and PDB names of the atoms that participate in the protein modification interactions. We will update this file with new protein modifications as they are identified in the PDB and added to RESID.

### 2.3 Identification of protein modifications in 3D structures

We devised different approaches for identifying protein modifications in each of the three categories.

#### 2.3.1 Identification of modified residues

Each modified residue is a unique component with a 3-character alphanumeric code in the worldwide PDB (wwPDB) Chemical Component Dictionary (http://www.wwpdb.org/ccd.html). In each of these components the parent corresponds to 1 of the 20 canonical amino acids. The corresponding entry in the aforementioned XML file recorded the 3-character code of each modified residue. For example, phosphoserine has a 3-character code SEP and its parent component serine has a 3-character code SER. Modified residues are included in the sequences reported along with the 3D structures in the PDB. Therefore, modified residues in a 3D structure are simply identified by scanning its sequence residues and comparing their 3-character codes with those in the XML file.

#### 2.3.2 Identification of attachment modifications

An attachment modification involves a linkage between one amino acid residue in a protein chain and at least one other chemical component such as a non-polymeric residue, ligand, inhibitor or ion. For each attachment modification, the customized XML file records the 3-character codes of the chemical component(s), together with a pair of atoms that form a chemical bond between them. For example, N4-glycosyl-l-asparagine involves residues asparagine (ASN) and N-acetyl-d-glucosamine (NAG) linked by a covalent bond between atom ND2 on ASN and atom C1 on NAG. There are many metal-binding modifications in the PDB where a single metal ion, such as zinc (ZN), is coordinated by amino acid residues such as histidine (HIS) or cysteine (CYS). To identify an attachment, we scan for residues in a protein chain that are within close proximity of components with 3-character codes specified in the XML file and check if they form a chemical bond between the listed atoms. Whether two atoms form a chemical bond is determined by checking the distance between the atoms against a threshold distance corresponding to the sum of their covalent radii plus a tolerance of error (0.4 Å by default).

#### 2.3.3 Identification of cross-links

A cross-link involves two or more amino acid residues in protein chains linked by covalent linkages. Similar to the attachment modifications, the customized XML file records all involved amino acid residues and pairs of atoms that form chemical bonds. For example, a disulfide bond involves two cysteine (CYS) residues linked to each other by their SG atoms. Identification of cross-links to non-polymer residues follows the same strategy as modified residues, since they have special 3-letter codes in the PDB. For canonical residue cross-links, we identify them by matching all involved residues, and atom pairs forming chemical bonds in a given structure. The criterion used to determine a chemical bond between two atoms is the same as in the case of attachment modifications.

### 2.4 Open source Java API

All source code and the protein modification XML file have been released as a BioJava package named BioJava-ModFinder, which is available at http://www.biojava.org. It is straightforward to identify protein modifications using the ModFinder application programming interface (API). A tutorial for the ModFinder API is available at (https://github.com/biojava/biojava-tutorial/blob/master/modfinder/README.md). The source code that generates the annotated sequence diagrams on the RCSB PDB website is also available from the BioJava repository in the rcsb-sequenceviewer project.

## 3 Results

### 3.1 Scanning the PDB

Using ModFinder, we scanned all 120 388 entries in the PDB (as of July 12, 2016). We identified 30 104 PDB entries with protein modifications classified by chemical processes in the PSI-MOD ontology, which includes 22 527 entries with cross-links (disulfide bonds and isopeptide bonds), 4631 glycoconjugated residues, 1577 oxidized residues, 1586 phosphorus containing residues, and 1235 acylated residues. The ModFinder module is run weekly after the update of the PDB database and the updated modifications are loaded as annotations into the RCSB PDB database. A current detailed breakdown of all the protein modifications is available online through the protein modification browser on the RCSB PDB website (Rose *et al.*, 2015). An up-to-date data file with protein modification annotation is available at https://cdn.rcsb.org/resources/protmod/protmod.tsv.gz.

### 3.2 Searching and browsing protein modifications in the PDB

**Fig. 1:**
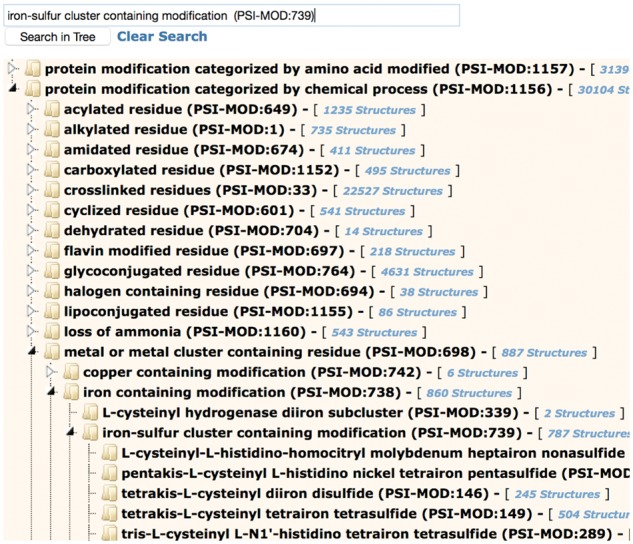
Protein Modification Browser on the RCSB PDB website showing the PSI-MOD hierarchy of protein modifications by amino acid modified and by chemical process. The iron-sulfur cluster containing modifications section of the tree has been expanded. By clicking on any part of the hierarchy, the user can retrieve associated PDB entries

Protein modification annotations are searchable through the ‘Advanced Search/Sequence Features’ option on the RCSB PDB website (Rose *et al.*, 2011, 2015). Search options include name, keyword, RESID ID, PSI-MOD ID, and PDB Chemical Component ID of the modification. A more convenient way to find protein modifications is through the protein modification browser, accessible by the ‘Browse by Annotations’ link at the top of the RCSB PDB website. This browser implements hierarchical navigation using two branches of the PSI-MOD ontology tree; by amino acid modified, and by chemical process (Fig. 1). When typing the name of a modification in the search box, suggested matches are displayed.

### 3.3 Mapping protein modifications to sequence and structure

**Fig. 2:**
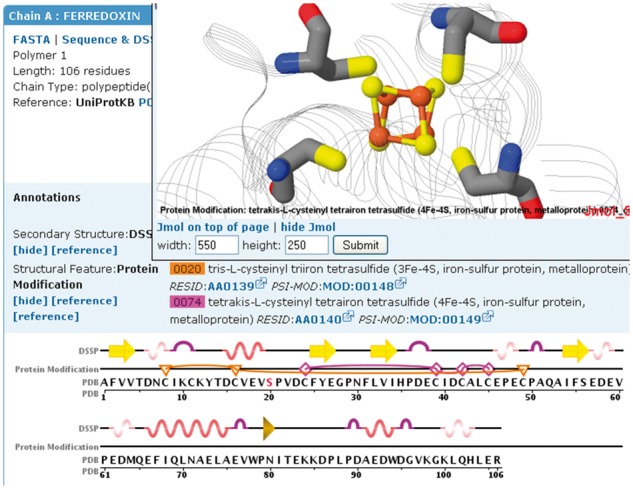
Protein modifications mapped onto the sequence and structure of ferredoxin I (PDB ID 1GAO, Chen *et al.*, 2002). The Protein Modification track highlights residues involved in two iron-sulfur clusters (3Fe-4S (F3S): triangles/lines and 4Fe-4S (SF4): diamonds/lines). The number of edges of the protein modification icon symbolizes the number of residues involved in the modification. The 4Fe-4S cluster is displayed in the Jmol structure window above the sequence display

The Sequence tab on each Structure Summary Page of the RCSB PDB website displays a diagram of the sequences in a selected PDB entry. The user can retrieve a number of other annotations, including protein modifications, and display them on the sequence by selecting it from a pull down menu. A 3D view of the protein modification can be seen by clicking on a specific modification on the sequence display, and viewing it in the accompanying Jmol display (Fig. 2). Currently, only modifications that belong to a single chain can be displayed.

## 4 Conclusion

We developed a novel software tool, ModFinder, for the identification and subsequent mapping of protein modifications to 3D structures in the PDB. Protein modifications are defined in an XML file that can be expanded as new modifications are discovered. ModFinder could also be easily extended to nucleic acid modifications. At present only modifications that are well curated by RESID and described by PSI-MOD and easily identifiable in PDB structures are included in the RCSB PDB. These modifications can be easily searched, browsed, and visualized on the RCSB PDB website. 
